# Some biological activities of *Epaltes divaricata* L. - an in vitro study

**DOI:** 10.1186/s12941-015-0074-4

**Published:** 2015-03-24

**Authors:** Leela Glorybai, Barathi Kannan K, Mariadhas Valan Arasu, Naif Abdullah Al-Dhabi, Paul Agastian

**Affiliations:** Department of Botany, N.K.R. Govt. Arts College (W), Namakkal, 637 001 India; Department of Plant Biology and Biotechnology, Loyola College, Chennai, 600034 India; Department of Botany and Microbiology, Addiriyah Chair for Environmental Studies, College of Science, King Saud University, Riyadh, 11451 Saudi Arabia

**Keywords:** Antimicrobial activity, α-glucosidase inhibition, Antioxidants activity, *Epaltes divaricata*, GC-MS analysis

## Abstract

**Background:**

Novel chemical molecules recovered from endangered medicinal plants have wide applications and have the potential to cure different diseases caused by microorganisms. The aim of this study was to investigate In vitro antimicrobial, α-glucosidase inhibition and antioxidant activity of different solvent extracts of *Epaltes divaricata* L.

**Methods:**

Antimicrobial activity of hexane, ethyl acetate and methanol extract of *Epaltes divaricata* was determined against bacteria and fungi using disc diffusion and microdilution method respectively. α-glucosidase inhibition, Total phenolic content (TPC), Reducing power activity, DPPH radical scavenging assay, hydroxyl radical scavenging activity, nitric oxide scavenging activity, superoxide scavenging activity and lipid peroxidation assay of plant extracts were performed according to standard protocol. Compound detection from the potential solvent extract was done through GC-MS analysis.

**Results:**

*Epaltes divaricata* ethyl acetate extracts (EDEa) (1.25 mg/disc) showed significant inhibition for *E. lentum* (23 mm), *E. aerogenes* (18 mm), *P. fluorescence* (15 mm) *and A. baumanii* (15 mm). Minimum inhibitory concentration (MIC) of EDEa was found to be 31.25 μg/ml, 62.5 μg/ml and 62.5 μg/ml against *A. flavus*, *A. niger* and *T. rubrum* respectively. EDEa showed more α-glucosidase inhibition and antioxidant activity compared to hexane and methanol. EDEa showed 50% α-glucosidase inhibition at the concentration of 525.20 ± 2.37 μg/ml. The TPC of EDEa was 412.0 ± 2.21 mg of catechol equivalents/g extract. EDEa showed great scavenging activity on 2,2-diphenyl-picrylhydrazyl (DPPH) (IC50 560 ± 2.02 μg/ml), hydroxyl (IC50 314.75 ± 2.56 μg/ml), nitric oxide (IC50 648.20 ± 2.09 μg/ml) and superoxide (IC50 361.14 ± 1.45 μg/ml) radicals, as well as high reducing power. EDEa also showed a more suppressive effect on lipid peroxidation. Using Antioxidant β-carotene linoleate method, the scavenging values of EDEa was significantly lower than BHT. GC-MS analysis of EDEa showed maximum amount of 2-butenamide, N-(4-fluorophenyl)-3-methyl trans-cinnamyl tiglate silane and trichlorocyclohexyl silane (36.86%).

**Conclusion:**

The results obtained in this study clearly indicate that EDEa can be used as a natural antimicrobial, α-glucosidase inhibition and antioxidant agent.

## Background

*Epaltes divaricata* (L.) (Family: Compositae), a plant used in traditional Ayurvedic medicine, is found in Sri Lanka, India, Myanmar, Java and China. It is used to alleviate jaundice, urethral discharges and acute dyspepsia, and is also regarded as a diaphoretic, diuretic and a stimulating expectorant. Currently, there has been a marked rise in scientific interest in the pharmacological activities of medicinal plants, particularly in relation to folklore medicine [1]. In recent years, methicillin and multiple antibiotic drug resistant strains of *Staphylococcus aureus* (MRSA) have caused outbreaks in hospital related infections throughout the world, and the WHO puts the international prevalence of nosocomial infections at 9%. Across the world, infectious disease is the primary cause of death, accounting for approximately one-half of all fatalities in tropical countries; infectious disease mortality rates are all increasing in developing countries and between 1981 and 1992, deaths from infectious disease increased to some 58% [2]. These negative health trends obviously point out the need for renewed interest and novel strategies for the prevention and treatment of infectious diseases focused on the development of new antimicrobials [3].

Antioxidants are substances that delay or inhibit oxidative damage to a target molecule. Antioxidant molecules react with single free radicals and can neutralize free radicals by donating one of their own electrons, thereby ending the carbon-stealing reaction. Antioxidants also act as scavenger and so prevent cell and tissue damage and cells provide defense against excessive free radicals by initiating preventative and repair mechanisms, repair mechanisms and physical and antioxidant defenses. A wide variety of plant constituents have been shown to be free-radical scavengers [4] a fact that has encouraged the work described here, in which solvent extracts of the medicinal herbs have been evaluated for both antimicrobial and antioxidant activity [5,6]. *Epaltes divaricata* L. is a seasonal medicinal plant occurs only during the month December, not much explored for its bioactivities; very scanty work has been carried out in the test plant *Epaltes divaricata (L.)*. Hence the present study, is taken up to investigate the solvent extracts for antimicrobial, antioxidant, anti-diabetic followed by identification of active compounds using GC-MS.

## Materials and methods

### Chemicals and reagents

DPPH (1,1-diphenyl,2-picrylhydrazyl), NBT (nitro blue tetrazolium), NADH (nicotinamide adenine dinucleotide phosphate reduced), PMS (phenazine methosulphate), TCA (trichloro acetic acid), ferric chloride and BHT (butylated hydroxyl toluene) were obtained from Sigma chemical co., USA. Ascorbic acid was obtained from SD fine chem. Ltd., Biosar, India. b-Carotene, ferrozine, folin– phenol reagent and Tween 40 were purchased from Hi-Media Pvt. Ltd. Mumbai, India. All the other chemicals were of analytical grade.

### Collection of *Epaltes divaricata* L

*Epaltes divaricata* L. plants were collected on the banks of Oragadam fresh water lake near Chennai, Tamil Nadu, India. *Epaltes divaricata* L. was identified and authenticated by Dr. J. Jeyajothi (Taxonomist), Department of Plant Biology and Biotechnology, Loyola College, Chennai, India. A voucher specimen (LCH-71) was deposited at the Loyola College Herbarium (LCH) in Loyola College, Chennai.

### Preparation of plant extracts

Healthy, disease-free whole plants of *Epaltes divaricata* L. were washed thoroughly, shade dried in room temperature and grounded into a powder. The powder was sequentially soaked in hexane, ethyl acetate and methanol for 72 h respectively with intermittent shaking [7]. After 72 h, the solution was filtered and the filtrate was concentrated under reduced pressure using a rotary vacuum evaporator; the crude extracts were then collected and stored at 4°C in an air tight container until further use.

### Assessment of antimicrobial activity

#### Test organisms

The following bacterial cultures were used to test antibacterial test- *Staphylococcus aureus* (MRSA), *Staphylococcus aureus* (Methicillin sensitive), *Eubacterium lentum, Bacillus subtillis, Enterobacter aerogenes, Vibrio fishcherii, Acinetoactor baumanii* (ICMR), *E.coli* (ATCC 25922), *Erwinia amylovora* (MTCC 2760), *Pseudomonas fluorescence, Klebsiella pneumonia.* The following fungal cultures were used for antifungal test- *Aspergillus flavus*, *Botrytis cinerea, Curvularia lunata* 46/01, *Aspergillus niger* MTCC 1344, *Trichophyton rubrum* 57/01 and *T. mentagrophytes* 66/01. All cultures were obtained from IMTECH, Chandigarh, India and the clinical isolates were obtained from the Department of Microbiology, Christian Medical College, Vellore, Tamil Nadu, India.

### Disc diffusion method

Antibacterial assay was carried out using disc diffusion method [8]. Petri plates were prepared using 20 ml of sterile MHA (Hi-media, Mumbai). The test culture (100 μl of suspension containing 10^8^ CFU/ml bacteria) was swabbed on the surface of the solidified media and allowed to dry for 10 minutes. Sterile discs (6 mm diam.) were impregnated with 25 μl (2.5 mg) of hexane, ethyl acetate and methanol extracts of the test plants. The discs were then dried at 37°C in laminar air flow chamber before use. Streptomycin (10 μg/disc) was used as a positive control. Plates were incubated overnight at 37°C for the measurement of zone of inhibition (in mm) around the disc. All experiments were repeated in triplicate.

### Antifungal assays using broth micro dilution method

The filamentous fungi were grown on SDA slants at 28°C for 7 days. The spores were collected using sterile double distilled water and stored in refrigerator until used. Antifungal activity was performed by following the method of Rejiniemon et al. 2014 [9]. The extracts were dissolved in water with 2% DMSO (concentration of the extract was 2 mg/ml). The initial test concentration was serially diluted two-fold in 96 well plate. Each well was inoculated with 5 μl of suspension containing approximately 10^4^ spore/ml. The antifungal agent, fluconazole was used as positive control and MIC was determined as the lowest extract concentration showing no visible fungal growth after incubation time.

### Determination of *in vitro* α-glucosidase inhibition and antioxidant assays

#### α-glucosidase inhibition activity

In order to investigate the inhibitory activity of solvent extracts of the test plants, an *in vitro* α-glucosidase inhibition test was performed. α-glucosidase is obtained from yeast and is used extensively as a screening material for α-glucosidase inhibitors. Since this assay does not always correlate with results obtained using mammals, we used mouse small-intestine homogenate as α-glucosidase solution in order to reflect better *in vivo* response. Enzyme inhibition was measured using a method slightly modified version of the method of Dahlqvist [10]. After fasting a mouse for 20 h, the small intestine was cut, rinsed with ice-cold saline, and homogenized with 12 mL of maleate buffer (100 mM, pH 6.0). The homogenate was then used as the α-glucosidase solution. The assay mixture consisted of 100 mM maleate buffer (pH 6.0), 2% (w/v) each sugar substrate solution (100 μl), and the sample extract (200–1000 μg /mL). Acarbose was used as standard for α- glucosidase inhibitor. The mixture was pre-incubated for 5 min at 37°C, and the reaction was initiated by adding the crude α-glucosidase solution (50 μl) to it, followed by incubation for 10 min at 37°C. The glucose released in the reaction mixture was determined by GOD-POD method; and absorbance was read at 505 nm. The rate of carbohydrate decomposition was calculated as a percentage ratio relative to the amount of glucose obtained when the carbohydrate was completely digested. The inhibition percentage was calculated using the following formula:

$$ \begin{array}{l}\mathrm{Inhibition}\ \left(\%\right) = \Big[\left(\mathrm{amount}\ \mathrm{of}\ \mathrm{glucose}\ \mathrm{produced}\ \mathrm{b}\mathrm{y}\ \mathrm{the}\ \mathrm{positive}\ \mathrm{control}\right)\ \\ {}\kern6em \hbox{--}\ \left(\mathrm{amount}\ \mathrm{of}\ \mathrm{glucose}\ \mathrm{produced}\ \mathrm{b}\mathrm{y}\ \mathrm{the}\ \mathrm{addition}\ \mathrm{of}\ \mathrm{sample}\right)\ \\ {}\kern5.75em /\ \left(\mathrm{amount}\ \mathrm{of}\ \mathrm{glucose}\ \mathrm{produced}\ \mathrm{b}\mathrm{y}\ \mathrm{the}\ \mathrm{positive}\ \mathrm{control}\right)\Big] \times 100\end{array} $$

### Total phenolic content (TPC)

Total phenolic content was determined using the Folin–Ciocalteau method [11] with some modifications. The sample (0.1 ml of a 200–1000 μg/ml solution), 1.9 ml distilled water and 1 ml of Folin–Ciocalteau’s reagent were added in a tube, and then 1 ml of 100 g/L Na_2_CO_3_ was added. The reaction mixture was incubated at 25°C for 2 h and the resultant absorbance of the mixture was read at 765 nm. The sample was tested in triplicate and a calibration curve with six data points for catechol was obtained. The results were compared to a catechol calibration curve and the total phenolic content of extracts was expressed as mg of catechol equivalents per gram of extract.

### Reducing power activity

The reducing power of extracts was determined according to Yen and Duh [12]. A range of concentrations of solvent crude extracts (200–1000 μg/mL) were mixed with 2.5 ml of phosphate buffer (200 mM, pH 6.6) and 2.5 ml of 1% potassium ferricyanide. The mixtures were incubated for 20 min at 50°C. After incubation, 2.5 ml of 10% trichloroacetic acid was added to each mixture followed by centrifugation at 3000 rpm for 10 min. The upper layer (5 ml) was mixed with 5 ml of distilled water and 1 ml of 0.1% ferric chloride. The absorbance of the resultant solution was measured at 700 nm and was compared with standard BHT absorbance.

### DPPH radical scavenging assay

DPPH quenching ability of solvent extracts of the test plant were measured according to Hanato *et al.* [13]. A methanol DPPH solution (0.15%) was mixed with serial dilutions (200–1,000 μg/ml) of the test plant extracts and the absorbance was read at 515 nm after 10 min. The antiradical activity was expressed as IC_50_ (μg/ml) (the antiradical dose required to cause a 50% inhibition). Vitamin C was used as standard. The ability to scavenge the DPPH radical was calculated by the following formula: DPPH radical scavenging activity % = A_0_ ‐ A_1_/A_0_ × 100 Formula 1.

Where A_0_ is the absorbance of the control at 30 min and A_1_ is the absorbance of the sample at 30 min. All samples were analyzed in triplicate.

### Hydroxyl radical scavenging activity

The hydroxyl scavenging assay was performed as described by the method of Elizabeth and Rao [14] with minor changes. All solutions were prepared freshly. One milliliter of the reaction mixture contained 100 μl of 28 mM 2-deoxy-2-ribose (dissolved in phosphate buffer, pH 7.4), 500 μl solution of various concentrations of solvent extracts of test plants (200–1000 μg/ml), 200 μl of 200 μM FeCl_3_ and 1.04 mM EDTA (1:1 v/v), 100 μl H_2_O_2_ (1 mM) and 100 μl ascorbic acid (1 mM). After an incubation period of 1 h at 37°C the extent of deoxyribose degradation was measured by the TBA reaction. The absorbance was read at 532 nm against the blank solution. Vitamin C was used as a positive control. The scavenging activity was calculated using formula (1).

### Nitric oxide scavenging activity

Sodium nitroprusside in an aqueous solution at physiological pH spontaneously generates nitric oxide; it interacts with oxygen to produce nitrite ions, which can be estimated by the use of Griess Illosvoy reaction [15]. Here, Griess Illosvoy reagent was modified using naphthyl ethylene diamine dihydrochloride (0.1% w/v) instead of 1-naphthylamine (5%). The reaction mixture (3 ml) containing sodium nitroprusside (10 mM, 2 ml), phosphate buffer saline (0.5 ml) and different concentration of solvent extracts of the test plants (200–1000 μg/ml) or standard solution (0.5 ml) were incubated at 25°C for 150 min. After incubation, 0.5 ml of the reaction mixture containing nitrite was pipetted and mixed with 1 ml of sulphanilic acid reagent (0.33% in 20% glacial acetic acid) and allowed to stand for 5 min for completing diazotization. Then, 1 ml of naphthyl ethylene diamine dihydrochloride (1%) was added, mixed and allowed to stand for 30 min. A pink colored chromophore was formed in diffused light. The absorbance of these solutions was measured at 540 nm against the corresponding blank. Ascorbic acid was used as standard. The scavenging activity was calculated using the formula (1).

### Superoxide scavenging activity

Superoxide scavenging activity of solvent extracts from the test plant was determined by monitoring the competition of those with NBT for the superoxide anion generated by the PMS–NADH system [16]. Superoxide radicals were generated in 1 ml of 20 mM Tris –HCl buffer (pH 8.0) containing 0.05 mM nitroblue tetrazolium (NBT), 0.01 mM phenazine metho sulphate (PMS) and different concentrations (200 – 1000 μg/ml) of extracts from test plants and were preincubated for 2 min. The reaction was initiated by the addition of 0.078 mM NADH. Blue chromogen, formed due to NBT reduction was read at 560 nm. Results were expressed as percentage of inhibition of superoxide radicals. Vitamin C was used as positive control. The scavenging activity was calculated using formula (1).

### Lipid peroxidation assay

The inhibition effect of solvent extract made from the test plant on lipid peroxidation was determined according to the thiobarbituric acid method. FeCl_2_ – H_2_O_2_ was used to induce the liver homogenate peroxidation [17]. In this method, 0.2 ml of extracts (200–1000 μg/ml) was mixed with 1.0 ml of 1% liver homogenate (each 100 ml homogenate solution contains 1.0 g rat liver), 50 μl of FeCl_2_ (0.5 mM) and H_2_O_2_ (0.5 mM) were added. The mixture was incubated at 37°C for 60 min, then 1.0 ml of trichloroacetic acid (15%) and thiobarbituric acid (0.67%) was added and the mixture was heated up in boiling water for 15 min. The absorbance was recorded at 532 nm. Ascorbic acid was used as the positive control. The percentage of inhibition effect was calculated according to formula (1).

### Antioxidant activity using β-carotene linoleate model system

The antioxidant activity of solvent extracts of the test plant were evaluated by β -carotene linoleate model [17]. A solution of β-carotene was prepared by dissolving 2 mg of β -carotene in 10 ml of chloroform. This solution (2 ml) was pipetted out into a 100 ml round-bottom flask. After the removal of chloroform under vacuum, 40 mg of purified linoleic acid, 400 mg of Tween 40 emulsifier and 100 ml of aerated distilled water were added to the flask with vigorous shaking. Aliquots (4.8 ml) of this emulsion were transferred into different test tubes containing different concentrations of the extracts (200–1000 μg/ml). As soon as the emulsion was added to each tube, the zero time absorbance was measured at 470 nm. The tubes were then placed at 50°C in a water bath. Measurement of absorbance was continued until the colour of β -carotene disappeared; a blank devoid of β -carotene, was prepared for background subtraction. BHA was used as positive control. Antioxidant activity (AA) was calculated using the following equation; AA = (β-carotene content after 2 h of assay/initial β-carotene content) ×100.

### GC –MS analysis

GC-MS analysis of the ethyl acetate extracts found to be better than other solvents of *Epaltes divaricata* L. was performed using a perkin Elmer GC clarus 500 system comprising AOC-20i auto-sampler and a Gas chromatograph interfaced to a Mass spectrometer (GC-MS) equipped with a Elite-5MS (5% Diphenyl/95% Dimethyl Poly Siloxane) fused capillary column (30 × 0.25 μm 1D × 0.25 μm dF). For GC-MS detection, an electron ionization system was operated in electron impact mode with ionization system operated in electron impact mode with ionization energy of 70ev. Helium gas (99.999%) was used as carrier gas at a constant flow rate of 1 ml/min, and an injection volume of 2 μl was employed (split ratio of 10:1). The relative percentage amount of each component was calculated by comparing its average peak area to the total areas. The mass detector used in this analysis was Turbo-Mass Gold-Perkin Elmer and the software adopted to handle mass spectra and chromatograms was a Turbo-Mass ver 5.2. GC-MS was conducted using the database of central electrochemical research institute characterization and measurement laboratory having more than 62,000 patterns. The spectrum of the unknown components was compared with the spectrum of known components stored in the data bank, Central Electrochemical Research Institute Characterization and Sargam metals, Chennai, India.

### Statistical analysis

The data were analyzed and expressed as means ± SD. The IC_50_ values were calculated from linear regression analysis. Results were processed using Microsoft Excel (2007).

## Results

### Antibacterial activity of *Epaltes divaricata*

The results of antimicrobial activity of ethanolic extract of *Epaltes divaricata* L. against various human pathogens are listed in the Table 1. The effect of EDEa extracts against various microbes and their biopotency were qualitatively and quantitatively assessed by determining the presence or absence of inhibition zones and zone diameters. Ethyl acetate extracts of the test plants were screened against reference cultures and clinical isolates. Zones of inhibition of the crude extracts were noted. The results showed that the ethyl acetate extract of *Epaltes divaricata* L. dose inhibits the growth of both bacteria (1.25 μg/disc) and fungi (least 31.5 μg/μl). Of the three different solvent (hexane, ethyl acetate and methanol) extracts tested for antimicrobial activity against the ATCC and MTCC reference cultures, most of the gram positive and gram negative bacteria were inhibited at different levels. With reference to gram positive bacteria tested, *Staphylococcus aureus* (MRSA) (13–15 mm) and *Eubacterium lentum* (19-23 mm) (Table 1) were affected by all the solvent extracts of test plant. Higher activity was found in ethyl acetate extracts followed by methanol and least activity found in hexane extracts. With reference to gram negative bacteria, with exception of *Klebsiella pneumoniae* (ATCC 15380) all the tested bacteria were affected significantly by all the solvent systems used for the plant studied. *Enterobacter aerogenes* (MTCC111), *Acenetobacter baumanii* (ICMR) and *Psuedomonas fluorescence* were affected significantly by all the solvent extracts of the tested plant. *Erwinia amylovora* (MTCC 2760) affected differently using different solvents of the test plant. Moderate activity was found against *Vibrio fishcherii* and *E.coli* in all tests.

**Table 1:** **Antibacterial activities of crude extracts of** ***Epaltes divaricata*** **L. values are the average of three different experiments measuring the zone of inhibition (mm)**

**Name of the pathogen**	**Antibactrerial activity (mg/ml)**			**Standard**
	**Zone of inhibition (mm)**			
	**Hexane**	**Ethyl acetate**	**Methanol**	
*Staphylococcus aureus*	9	14	15	26
*Staphylococcus aureus* (Methicillin sensitive)	-	-	-	13
*Eubacterium lentum*	10	23	19	28
*Bacillus subtillis*	-	12	13	14
*Enterobacter aerogenes*	8	18	13	22
*Vibrio fishcherii*	-	14	15	19
*Acinetobactor baumanii* (ICMR)	-	15	16	21
*E.coli* (ATCC 25922)	-	11	10	17
*Erwinia amylovora* (MTCC 2760)	-	10	-	16
*Psuedomonous fluorescence*	10	15	16	20
*Klebsiella pneumonia*	-	-	-	16

### Antifungal activity of *Epaltes divaricata L.*

Assay of the antifungal activity of the different solvents (ethyl acetate, methanol and hexane) extracts of medicinal plant *Epaltes divaricata* L. was carried out using micro broth dilution method. The MIC was significantly lower in ethyl acetate extracts that inhibits *A. flavus* (31.25 μg/ml), *A. niger, T. rubrum* (62.5 μg/ml) followed by methanol extracts (125 μg/ml). Hexane extracts of this plant requires more or less higher concentration of extract to inhibit the test fungus (Table 2).

**Table 2:** **Antifungal activities of crude extracts of** ***Epaltes divaricata*** **L values are the average of three different experiments measuring the μg/ml**

**S.No**	**Tested fungi**	**Hexane (μg/ml)**	**Ethyl acetate (μg/ml)**	**Methanol (μg/ml)**	**Fl (μg/ml)**
1	*Curvularia lunata* 46/01	250	125	125	125
2	*T. rubrum* 57/01	250	62.5	250	250
3	*T. mentagrophytes* 66/01	250	125	125	250
4	*Botrytis cinerea*	250	250	125	250
5	*Aspergillus flavus*	250	31.2	125	62.5
6	*Aspergillus niger* MTCC 1344	250	62.5	250	250

Fl : Fluconazole, an antifungal agent.

### *In vitro* α-glucosidase inhibition and antioxidant assays of *Epaltes divarica*ta (L.)

#### α-Glucosidase inhibition

The results for α-glucosidase inhibition assay of EDEa extract and acarbose are shown in Table 1. The concentration for 50% inhibition of ethyl acetate and acarbose was found to be 624.15 ± 2.37 and 260.32 ± 1.23 μg/ml respectively. The hexane and ethyl acetate extracts showed less inhibition compared to methanol extract (Table 3).

**Table 3:** **α-Glucosidase inhibiton of extracts of** ***Epaltes divaricata*** **L.**

**Sample**	**Concentration (μg/ml)**	**% of Inhibition**	**IC50 (μg /ml)**
	200	14.28	982 ± 2.04
	400	23.08	
Hexane	600	31.3	
	800	43.7	
	1000	49.9	
	200	29.2	624.15 ± 1.97
	400	37.25	
E.acetate	600	49.52	
	800	58.4	
	1000	69.5	
	200	5.33	2204 ± 2.56
	400	10.1	
Methanol	600	14.57	
	800	18.75	
	1000	23.23	
	200	45.91	260.32 ± 1.23
	400	61.14	
Acarbose	600	91.75	
	800	92.76	
	1000	93.93	

### Total phenolic content

**Figure 1:**
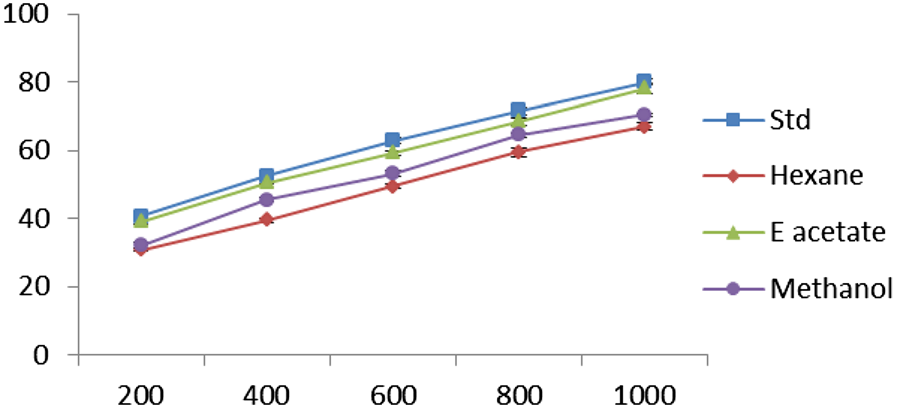
**Total phenolic content effect of different concentrations (200–1000 μg/ml) of** ***Epaltes divericata*** **L** ***.*** **hexane, ethyl acetate, methanol extracts and vitamin C.** Each value represents the mean ± SEM of triplicate experiments.

The total phenolic content of EDEa and catechol were found to be 412.0 ± 2.21 and 365.12 ± 2.23 μg/ml respectively (Figure 1).

### Reducing power

**Figure 2:**
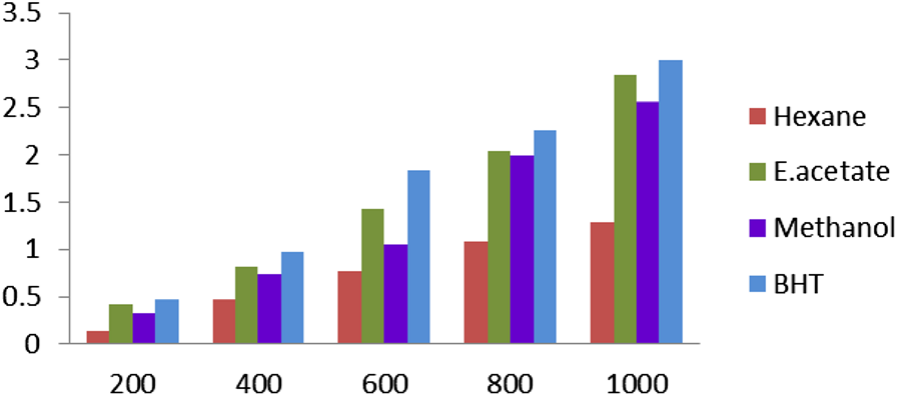
**Reductive ability of different concentrations (200–1000 μ/ml) of** ***Epaltes divericata*** **L** ***.*** **hexane, ethyl acetate, methanol extracts and BHT.** Each value represents the mean ± SEM of triplicate experiments.

Reductive capabilities of hexane, ethyl acetate and methanol extracts of *E. divaricata* L. compared the standard butylate dhydroxy toluene. The reducing power of EDEa was markedly more potent than the hexane and ethyl acetate extracts. The activity of the extract increased with increasing quantity of sample. Although the effect was less than the standard butylated hydroxytoluene (BHT), the plant extract was active in reducing Fe^3+^ ions (Figure 2).

### DPPH radical scavenging activity

**Figure 3:**
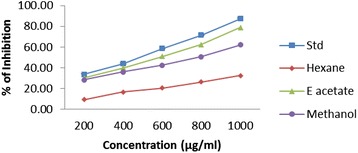
**DPPH scavenging effect of different concentrations (200–1000 μg/ml) of** ***Epaltes divericata*** **L** ***.*** **hexane, ethyl acetate, methanol extracts and vitamin C.** Each value represents the mean ± SEM of triplicate experiments.

EDEa exhibited a significant dose dependent inhibition of DPPH activity exhibiting 50% inhibition (IC_50_) at a concentration of 560 ± 2.02 μg/ml. The results are presented in Figure 3. The IC_50_ value of vitamin C was 465.1 ± 2.15 μg/ml.

### Hydroxyl radical scavenging assay

**Figure 4:**
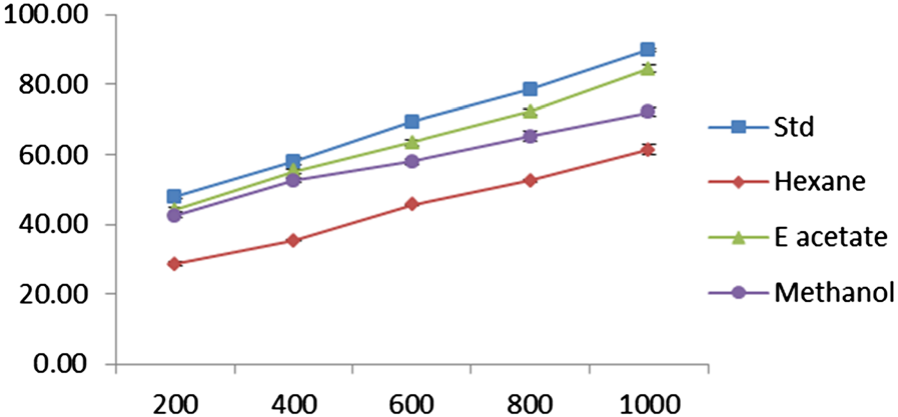
**Hydroxyl radical scavenging effect of different concentrations (200–1000 μg/ml) of** ***Epaltes divericata*** **L. hexane, ethyl acetate, methanol extracts and vitamin C.** Each value represents the mean ± SEM of triplicate experiments.

To attack the substrate deoxyribose hydroxyl radicals were generated by reaction of ferric-EDTA together with H_2_O_2_ and ascorbic acid. The results for hydroxyl scavenging assay are shown in Figure 4. The EDEa extract concentrations for 50% inhibition were found to 314.75 ± 2.56 used with ethyl acetate extract and vitamin C. Hexane and methanol extracts showed less effect.

### Nitric oxide radical inhibition assay

**Figure 5:**
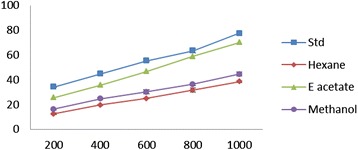
**Nitric oxide scavenging effect of different concentrations (200–1000 μg/ml) of** ***Epaltes divericata*** **L** ***.*** **hexane, ethyl acetate, methanol extracts and vitamin C.** Each value represents the mean ± SEM of triplicate experiments.

The scavenging of nitric oxide by EDEa was increased in a dose-dependent manner as shown in Figure 5. At a concentration of 648.20 ± 2.09 μg/ml of extract 50% of nitric oxide generated during incubation was scavenged. The IC_50_ value of vitamin C was 505.12 ± 1.98 μg/ml.

### Superoxide scavenging activity

**Figure 6:**
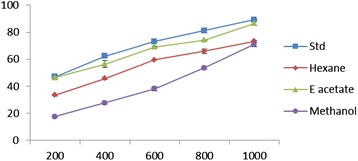
**Superoxide scavenging effect of different concentrations (200–1000 μg/ml) of** ***Epaltes divericata*** **L. hexane, ethyl acetate, methanol extracts and vitamin C.** Eachvalue represents the mean ± SEM of triplicate experiments.

The superoxide anion derived from dissolved oxygen by phenazine methosulphate/NADH coupling reaction reduces nitro blue tetrazolium. The decrease in absorbance at 560 nm with the plant extract thus indicates the consumption of superoxide anion in the reaction mixture. As mentioned in Figure 6, the ethyl acetate extract as well as vitamin C showed scavenging activity; IC_50_ values were 361.14 ± 1.45 μg/ml and 200.08 ± 2.51 μg/ml respectively, whereas hexane and methanol extracts there was no significant scavenging activity.

### Lipid peroxidation assay

**Figure 7:**
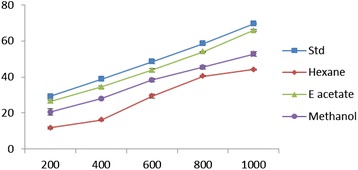
**Lipid peroxidation effect of different concentrations (200–1000 μg/ml) of** ***Epaltes divericata*** **L** ***.*** **hexane, ethyl acetate, methanol extracts and vitamin C.** Each value represents the mean ± SEM of triplicate experiments.

Activity of extracts on lipid peroxidation is shown in Figure 7. Addition of Fe^2+^/ascorbate to the liver microsomes cause increase in lipid peroxidation. EDEa extracts showed inhibition of peroxidation effect at all concentrations compared to hexane and methanol, which showed 50% inhibition effect at 703.32 ± 2.00 μg/ml. The IC_50_ value of vitamin C was 621.35 ± 1.88 μg/ml.

### Antioxidant using a β-carotene linoleate model system

**Figure 8:**
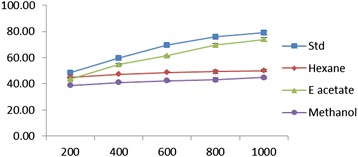
**Antioxidant activity of different concentrations (200–1000 μg/ml) of** ***Epaltes divericata*** **L. hexane, ethyl acetate, methanol extracts and vitamin C in the b carotene bleaching assay and butylated hydroxyl anisole (BHA).** Each value represents the mean ± SEM of triplicate experiments.

In the β-carotene linoleate system, β-carotene undergoes rapid discolouration in the absence of antioxidants. The addition of extracts to this system prevented the bleaching of β-carotene at different degrees. *Epaltes divaricata* L hindered the extent of β-carotene bleaching on a dose dependent manner for both hexane and ethyl acetate extracts. Based on 120 min reaction time (Figure 8), the ethyl acetate extract showed 50% inhibition at 320.10 ± 1.04 μg/ml and the value for BHA was 173.12 ± 2.28 μg/ml.

### GC-MS profiles of *Epaltes divaricata* (L.)

**Figure 9:**
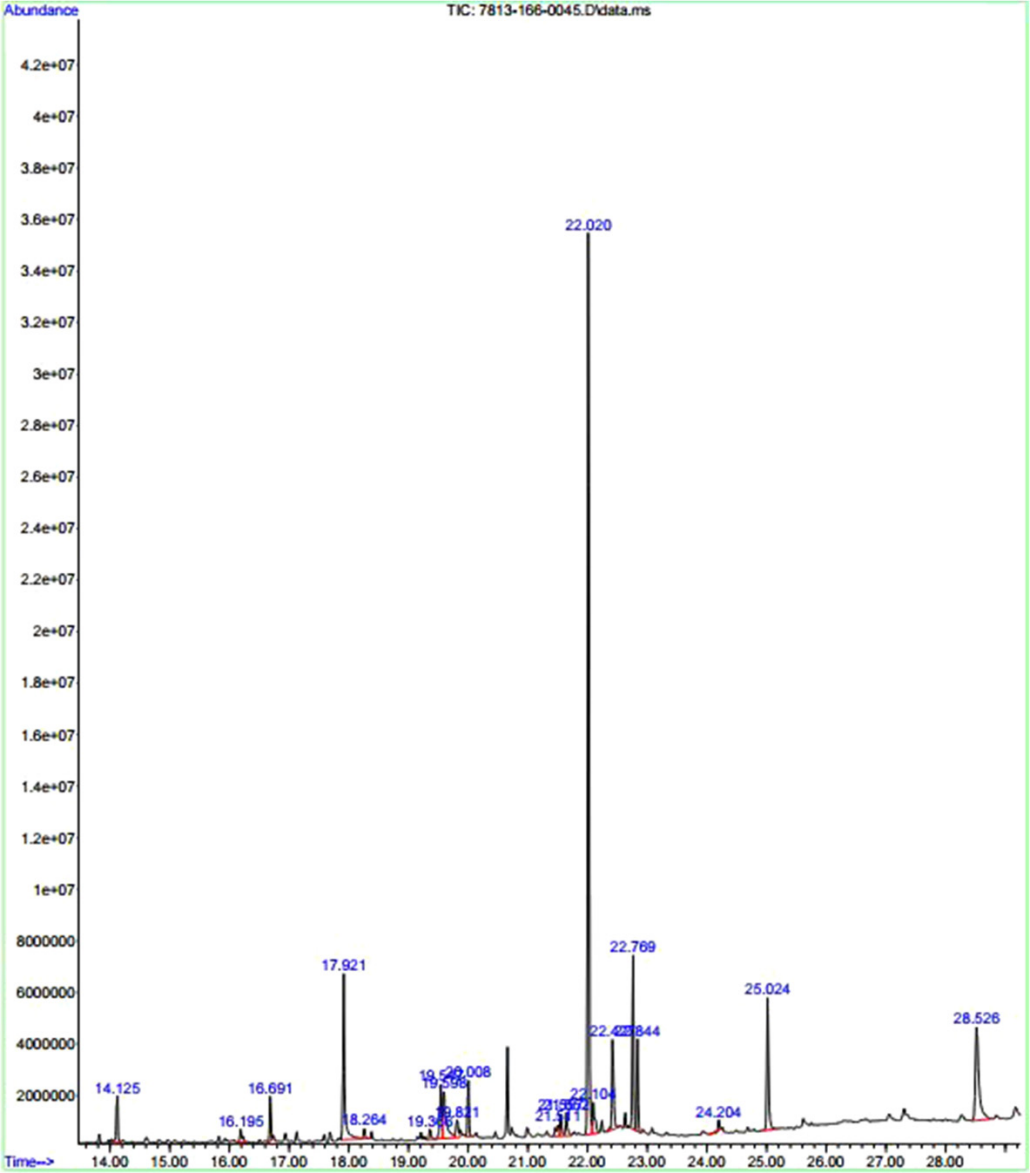
**GC – MS Chromatogram of Ethyl acetate extracts of** ***Epaltes divericata*** **L.**

From the activity results it was observed that the antioxidant and antimicrobial activity of the *Epaltes divaricata* L. was comparatively more in the ethyl acetate extract then other solvents (Figure 9). Ethyl acetate extracts of the test plants clearly showed the presence of diverse molecules. In *Epaltes divaricata* L. a total of 21 compounds were detected, out of which the maximum area was found 2-Butenamide, N-(4-fluorophenyl)—methyltrans cinnamyl tiglate silane, trichlorocyclohexyl silane (Table 4) with a value of 38.86% (Figure 9).

**Table 4:** **Activity of Phytocomponents identified in the ethyl acetate extracts of** ***Epaltes divaricata*** **L** ***. (*** **GC-MS Study)**

**S.No**	**Chemical name**	**Retention Time**	**% Area**
1	Neryl (S)-2-methylbutanoate 2,6-Octadien-1-ol, 3,7-dimethyl-, acetate, (Z)- Pentanoic acid, 3,7-dimethyl-2,6-ctadienyl ester, (E)	14.12	2.10
2	2-Cyclohexen-1-one, 4-hydroxy-3,5, 5-trimethyl-4-(3-oxo-1-butenyl)- Phenol, 4-methoxy-, acetate 5-Ethylcyclopent-1-enecarboxaldehyde	16.21	0.82
3	1,1′-Bicyclopentyl 3,4-Octadiene, 7-methyl Santolina epoxide	16.69	1.75
4	n-Hexadecanoic acid	17.91	8.17
5	Hexadecanoic acid, ethyl ester Octanoic acid, ethyl ester	18.26	0.57
6	Phytol 1-(3-Isobutyryl-bicyclo [1.1.1] pent −1-yl)-2-methylpropan-1-one 7-Oxabicyclo [4.1.0] heptane, 1,5-di methyl	19.36	0.63
7	9,12-Octadecadienoic acid (Z,Z)	19.54	2.63
8	9,12-Octadecadienoic acid (Z,Z) Cyclododecyne 9,12,15-Octadecatrienoic acid, (Z, Z,Z)	19.59	4.52
9	9,12-Octadecadienoic acid, ethyl ester 1,3,12-Nonadecatriene 9,17-Octadecadienal, (Z	19.81	1.19
10	Methanone, dicyclohexyl 1,2,4-Triazole, 5-cyclohexanecarbo xamido- 1-Propanone, 1-cyclohexyl	20.00	2.44
11	Tricyclo [4.3.1.1(3,8)] undecane-1-c arboxylic acid o-Anisaldehyde, semicarbazone Acetamide, N-[2-(4-methylphenoxy) ethyl	21.51	0.86
12	Isocitronellol 2-Hexene, 4,4,5-trimethyl Cyclohexane, bromo	21.56	1.17
13	Spiro [2,4,5,6,7,7a-hexahydro-2-oxo −4,4,7a-trimethylbenzofuran]- 7,2′-(oxirane) Neoclovene oxide Benzyl isobutyl ketone	21.64	1.20
14	2-Butenamide, N-(4-fluorophenyl)--methyltrans Cinnamyl tiglate Silane, trichlorocyclohexyl Silane	**22.02**	**38.86**
15	Lycorenan-7-one, 4,12-dihydro-5-hy droxy-1-methyl-9,10- [methylenebis(oxy)], (5.alpha.,12.beta.)-Furazan, 3- (dimethylaminomethylena mino)-4-(1,2,4-triazol-3-yl)- (E)-2-Isopropyl-5-methylphenyl 2-m ethylbut-2-enoate	22.09	2.11
16	Ar-tumerone 2-Butenamide, N-(4-fluorophenyl)--methyl- Methanone, dicyclohexyl	22.43	4.48
17	4,6-Dimethoxy-1-naphthaldehyde Benzofuro [3,2-d] pyrimidin-4(3H)-on e, 8-methoxy- 2-Pyrazolin-5-one, 4-acetyl-3-methyl-1-phenyl	22.76	6.90
18	Imidazole, 2-acetamido- 3-Ethyl-5-methyl-1-heptyn-3-ol 2-Butenoic acid, 2-methyl-, 2-prop enyl ester, (E)	22.83	3.51
19	Eicosane 2-methyltetracosane	24.20	0.30
20	Squalene	25.03	6.40
21	Stigmasterol 2-Pyridinamine, N-(4,5-dihydro-5-m ethyl-2-thiazolyl)-3-methyl	28.53	9.37

These “bold numbers” are the part of the IUPAC name of the particular compounds. The letters are written based on the functional group confirmation of the particular compound.

## Discussion

A number of researchers have commented on the Indian medicinal plants, [18-21]. They used a variety of solvents and have come to different conclusion, although they all agree that the phytoconstituents present in the plants, namely flavonoids, alkaloids tannins and triterpenoids provide exciting opportunities for the expansion of modern chemotherapies against a wide range of microorganisms [22]. In our *in vitro* studies, EDEa was shown to exhibit substantial inhibition of α-glucosidase and able to adsorb and neutralize free radicals, quenching singlet and triplet oxygen and decomposing peroxides. The increased antioxidant activity of the extracts *Epaltes divaricata* (L.) extracts appears to be due to the presence of phenolics. We suggest that *Epaltes divaricata* (L.) could potentially be used in the treatment of postprandial hyperglycemia. Based on the results of DPPH-radical assay, the ethyl acetate extracts of *Epaltes divaricata* (L.) exhibited a significant inhibitory activity against DPPH radical, whereas the ethyl acetae extract was found to have much less effect on free radical inhibition.

Plants contain many novel compounds with medicinal values which need further scientific evaluation. Free radicals are produced in aerobic cells due to consumption of oxygen during cell growth. Free radicals cause decrease in membrane fluidity, loss of enzyme receptor activity and damage to membrane protein leading to death [23]. These free radicals are involved in different disorders like aging, cancer, cardiovascular disease, diabetes, rheumatoid arthritis, epilepsy, as well as in the degradation of essential fatty acids; antioxidants helps in eluviation of the above disorders. Since the methanol extract of this plant showed the dose dependent antioxidant activity comparable to ascorbic acid, antioxidant agent might be developed from this plant for the treatment of above mentioned disorders associated with free radicals. Phenolic compounds containing free hydrogen have been shown to be largely responsible for antioxidant activity [24], as a result, the phenolic compounds of EDEa may be responsible for the plant’s antioxidant activity.

In general, higher concentrations of natural antimicrobial substances are required to inhibit bacteria in nutrient agar than in nutrient broth [25,26]. The antimicrobial activity was previously reported for other genus of *V. blumeoides* using 50 and 500 mg/L concentrations. Gram negative bacteria appear to be the least sensitive to the action of many other plant extracts and tested compounds [27]. In our study the ethyl extract of *Epaltes divaricata* (L.) exhibits significant activity against gram-positive bacteria similar to previous reports. The MIC of 125 μg /ml was showed against *Curvularia lunata* and *T. mentagrophytes* in order to determine antifungal activity*.*

Additionally the toxicity of some of the extracts/active ingredients was tested in a range of animal system which revealed that most of them had diverse side effects and toxic doses varied greatly [28-30]. One hundred and twenty one clinically useful prescription drugs have been derived from plants [31]. Surveys of plant medicinal usage by the American public have indicated an increase from 3% of population in 1991 to 37% in Brevoort [32]. Plants will continue to provide novel products as well as chemical models for new drugs in the future since the chemistry of the active extract from the majority of plant species has yet to be characterized. Compounds isolated from various plants and used for antimicrobial activity was well documented [33].

In general, phenolics and organic acids originating from plant materials are microbial decomposition products that accumulate in forest soil [34,35]. The antifungal activity exerted by phenolics and organic acids together may act on some microorganisms. *Candida albicans* can colonize or infect virtually all body sites because of its high adoptability to different post niches by the activation of appropriate sets of genes in response to complex environmental signals [36]. Kope and Fortin [37] reported that frequent morphological changes caused by antagonism were hyphal branching, bulging and swelling of cells, increase in septation and lysis. *Trichophyton rubrum* is the main agent isolated in superficial mycosis in Brazil, corresponding to almost 60% of all clinical cases [38]. Recently, *Candida* species associated candidemia have shifted from *Candida albicans* to non-albicans. The antifungal activity of *M. urundeuva* heartwood was detected using only 50 μg of lectin, a quantity much lower than 225 μg determined for a chitin binding lectin isolated from *Artocarpus* species on *Fussarium moniliforme* [39], revealing the high ability of heartwood lectin to inhibit fungal growth. The antifungal activity of lectins have been related to interfere with spore germination, probably very initial stage of process extending the latent period that precedes germination [40].

The solvent extracts of the test plants exhibited a range of antibacterial activity and may therefore prove promising in relation to the development of new antibiotics. Phytochemicals like phenolics can cause membrane disruption, form complex with cell wall, inactivate enzymes and cause substrate deprivation [41], and the in ingestion of carbohydrate rich diet causes elevation in blood glucose level by the rapid absorption of carbohydrates in the intestine aided by the action of glycoside hydrolases which breaks complex carbohydrates into absorbable monosaccharides. Thus use of glycosidase inhibitor such as α-glucosidase inhibitors would be a prospective therapeutic agent for the effective management of diabetes. α-Glucosidase inhibitors inhibit disaccharide digestion and impedes the postprandial glucose excursion to enable overall smooth glucose profile. Several α-glucosidase inhibitors have been isolated from medicinal plants for evaluation as an alternative drug with increased potency and lesser adverse effects than the existing drugs [42]. *Epaltes divaricata* shows a promising candidate by showing increased antimicrobial, α- glucosidase inhibition and antioxidant activity. The active extracts of ethyl acetate showed 2-Butenamide, N-(4-fluorophenyl)--methyltrans Cinnamyl tiglate Silane, trichlorocyclohexyl Silane and its derivatives would be a good candidate for eradication of human disease.

## Conclusion

To our knowledge, the present report is the novel investigation which demonstrated the effect of plant derived non-antibiotic compound directed against 10 bacteria and 7 fungi tested. The results indicate that phenolic and other related compounds are responsible for inhibition of microbes and antioxidants. 2-Butenamide, N-(4-fluorophenyl)--methyltrans Cinnamyl tiglate Silane, trichlorocyclohexyl Silane compounds revealed under GC-MS would be responsible for above mentioned bioactivity. These results are potentially relevant to the search for novel antimicrobials and antidiabetics.
